# High‐efficiency genome‐editing, transgene evaluation, and antimicrobial efficacy testing using *Citrus medica* L. hairy roots

**DOI:** 10.1111/tpj.70745

**Published:** 2026-02-18

**Authors:** Aditya Kulshreshtha, Manikandan Ramasamy, Sonia Irigoyen, Carmen S. Padilla, Chi‐Kuan Tu, Kinnie Laughlin, James Borneman, Kranthi K. Mandadi

**Affiliations:** ^1^ Texas A&M AgriLife Research and Extension Center Weslaco TX USA; ^2^ Department of Plant Pathology and Microbiology Texas A&M University College Station TX USA; ^3^ Department of Microbiology & Plant Pathology University of California Riverside CA USA; ^4^ Institute for Advancing Health Through Agriculture Texas A&M AgriLife College Station TX USA

**Keywords:** antimicrobial peptide, *Candidatus* Liberibacter asiaticus (*C*Las), *Citrus medica* L., Huanglongbing (HLB) disease, *NONEXPRESSOR OF PATHOGENESIS‐RELATED GENE 3* (*NPR3*)

## Abstract

Huanglongbing (HLB) disease, associated with the fastidious bacterium *Candidatus* Liberibacter asiaticus (*C*Las), has a significant impact on citrus production worldwide. Conventional biochemical and genetic evaluation studies to identify potential disease resistance strategies have been mainly hindered due to the inability to culture *C*Las in a defined medium and the general recalcitrance of *Citrus* cultivars (grapefruits and oranges) to *Agrobacterium*‐mediated plant transformation. We previously demonstrated the utility of plant hairy roots to co‐cultivate *C*Las. In this study, we developed a hairy root transformation system using citron (*Citrus medica* L.), which is highly amenable to *Rhizobium*‐mediated hairy root transformation. The explant survival and hairy root transformation efficiencies were up to 100% and 73%, respectively, and transgenic roots can be attained in as little as 30–60 days. We demonstrate the utility of this citron‐based hairy root transformation for rapid CRISPR/Cas9‐mediated gene editing, transgene evaluation, and antimicrobial efficacy testing. The citron‐based hairy root transformation system will significantly help the research community to speed‐track the assessment of potential HLB disease resistance strategies.

## Significance Statement

Huanglongbing (HLB) or citrus greening disease threatens global citrus production, yet progress toward effective resistance strategies has been limited by the causal pathogen's unculturable nature and the difficulty of transforming citrus plants. This study introduces a highly efficient citron‐based hairy root transformation system that enables rapid gene editing, transgene testing, and antimicrobial evaluation. By accelerating functional studies and resistance screening, this platform provides a practical tool to advance citrus disease research and supports the development of sustainable HLB management solutions worldwide.

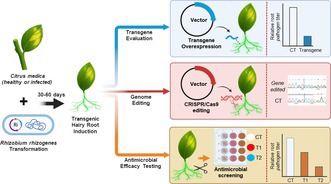

## INTRODUCTION

*Citrus* spp. is an economically important fruit crop and a valuable source of human nutrition, providing vitamins, minerals, and dietary fiber (Miles & Calder, 2021). However, huanglongbing (HLB) or citrus greening disease, putatively caused by a phloem‐limited fastidious bacterium, *Candidatus* Liberibacter asiaticus (*C*Las), significantly diminishes citrus productivity. It was first reported in China and has since become widespread across many citrus‐growing regions in Asia and the Americas (Gottwald, 2010). The HLB‐infected citrus plants typically exhibit stunted growth, weakened root and shoot systems, and asymmetrical leaf yellowing, referred to as blotchy mottle. The market value is also reduced as the infected plants produce small, unripe, and bitter fruits (Spreen et al., 2014).

The bacterium is transmitted from one plant to another by the Asian citrus psyllid (*Diaphorina citri*) and grafting of infected plant material. Inside the host, *C*Las colonizes the phloem tissues and blocks nutrient transport, leading to progressive decline and eventual tree death. HLB susceptibility is suggested to be induced by the secretion of Sec‐dependent effectors (SDEs) by the *C*Las bacterium in the phloem of host plants. SDEs suppress plant defense and help in bacterial colonization (Hu et al., 2025). Despite extensive research, no effective treatment or HLB‐resistant citrus varieties are currently available.

Since the bacterium resides intracellularly within phloem tissues, applying chemical treatments is difficult, as they cannot penetrate the infection site in effective concentrations. In addition, the pathogen cannot be cultured in artificial growth media, as it has a highly reduced genome, lacks many genes/components involved in metabolic pathways and integrated secretion systems, and plausibly relies on the host machinery and associated microbial species for survival and perpetuation (Merfa et al., 2019). These factors significantly impede both its pathological characterization and the screening of potential therapies (Yang & Ancona, 2022). Consequently, many indirect approaches have been explored to evaluate suitable therapies against HLB disease. These include growth‐inhibition assays using the culturable surrogate bacterium *Liberibacter crescens*, a *Sinorhizobium meliloti‐*based system to screen antimicrobials targeting *C*Las transcription factors (Barnett et al., 2019), antimicrobials testing in *C*Las‐infected psyllid tissues, and various *in planta* approaches, including hairy root assay, detached leaf assay, trunk injection, and root drenching (Kennedy et al., 2023). While these methods can identify many compounds, further evaluation is necessary to examine their effects on plant toxicity, associated beneficial microbial communities, and human health. Thus, current HLB management relies on integrated approaches including insecticide treatment to kill psyllid vectors, thermotherapy in young trees, trunk injection of antibiotics under specific regulatory protocols, and strengthening overall plant health through beneficial microbes, essential nutrients, maintaining nutrient‐rich soil, and minimizing abiotic stresses (Graham et al., 2024).

One promising strategy for treating HLB could be the use of *Agrobacterium*‐mediated stable genetic manipulations of plant defense or susceptibility genes. The method is advantageous as *Agrobacterium* introduces stable and inheritable genetic modifications. However, the *Agrobacterium*‐mediated transformation of woody plants, such as citrus, is challenging and can take multiple years to evaluate gene function (Conti et al., 2021). In a manner similar to *Agrobacterium*, *Rhizobium rhizogenes* can be used to study the functions of certain genes and the subcellular localization of proteins by inducing so‐called transgenic “hairy roots” (Ron et al., 2014). We previously demonstrated the utility of plant hairy roots for genetic evaluation in citrus, which is relatively faster (~4–6 months) (Irigoyen et al., 2020). We evaluated the functions of several heterologous genes, including broad‐spectrum antimicrobial peptides (AMPs) from spinach, genes involved in disease resistance (*NONEXPRESSOR OF PATHOGENESIS‐RELATED GENE1* and *NPR3*) in citrus and potato hairy roots (Irigoyen et al., 2020; Ramasamy et al., 2024). Moreover, we also demonstrated that stable citrus transgenic plants can be regenerated from hairy roots (Ramasamy et al., 2023). Despite the utility of *R. rhizogenes* in citrus biotechnology, cultivars such as grapefruits and oranges respond poorly, with lower hairy root transformation efficiency, and the duration of transformation remains longer (~90–120 days).

In this study, we report a robust citrus hairy root transformation system based on citron (*Citrus medica* L.), yielding up to 100% explant survival and transformation efficiencies of up to ~73% in as little as 30–60 days. We further demonstrate the utility of citron hairy roots for the rapid assessment of antimicrobial peptide efficacy, CRISPR/Cas9‐mediated loss‐of‐function studies, and high‐throughput chemical efficacy assays.

## RESULTS

### High‐efficiency hairy root transformation in citron

**Figure 1:**
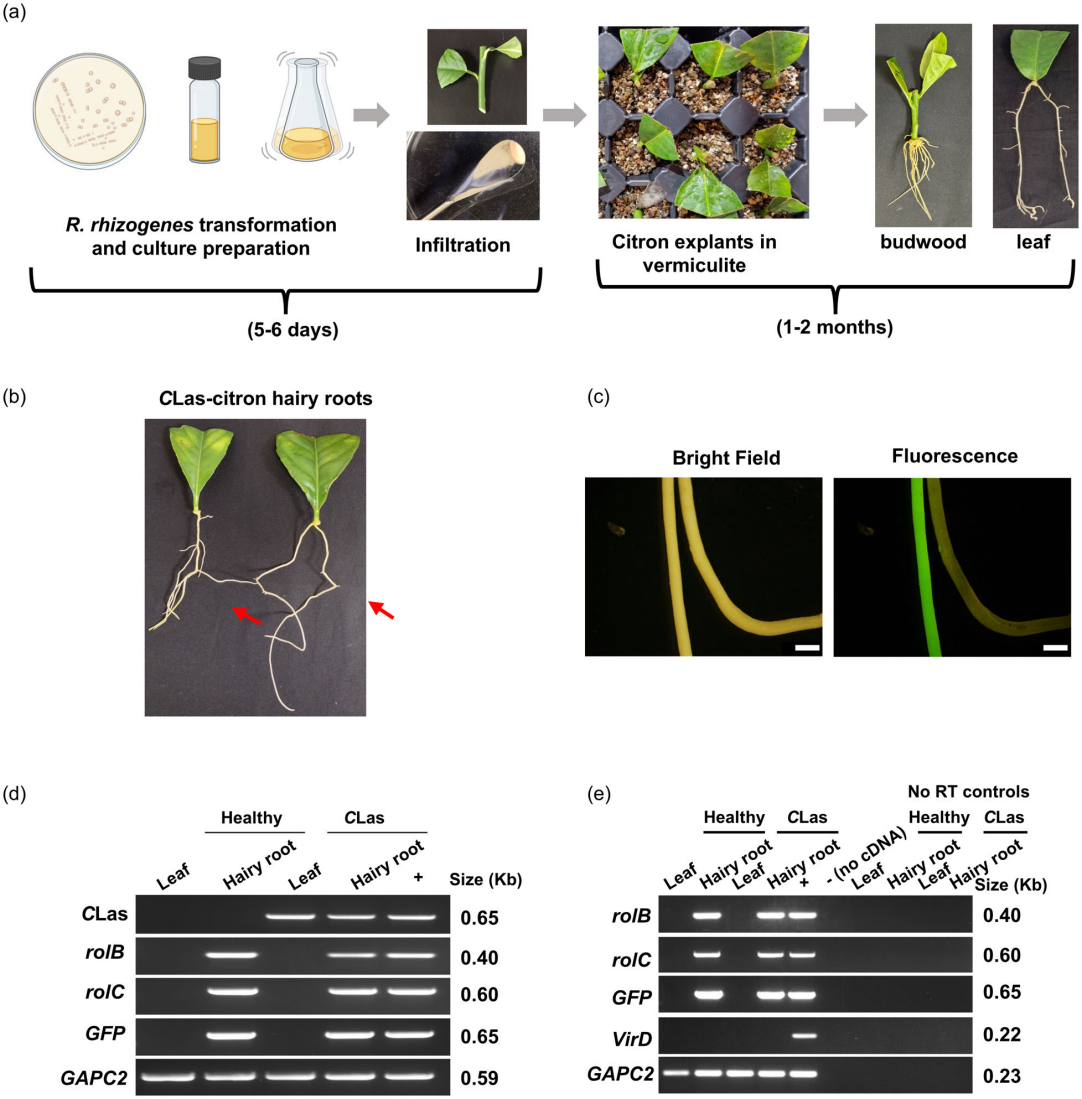
*Rhizobium rhizogenes*‐mediated hairy root transformation in citron. (a) Schematic representation of hairy root induction from citron budwood and petiole explants. (b, c) Hairy roots (shown by arrows) from *Candidatus* Liberibacter asiaticus (*C*Las)‐infected petioles and visual confirmation of GFP under a fluorescence microscope at 60 days post‐transformation. Scale bars 1 mm. (d) PCR‐based detection of *C*Las in citron leaf and hairy root samples. (e) Transgenic hairy roots were further validated by reverse transcription‐polymerase chain reaction (RT‐PCR) analysis to determine the expression of *GFP, rolB*, and *rolC*. An endogenous citrus *GAPC2* was used as an internal control for housekeeping gene expression. The PCR amplification of the *R. rhizogenes* virulence gene *VirD*, which is present outside the Ri T‐DNA region, and No RT‐PCR reactions were used to verify the absence of bacterial DNA contamination. “+” and “−” indicate positive and negative controls for respective PCR amplification. Raw agarose gel images used to prepare panels “d” and “e” are shown in Figure S2.

Here, we developed a robust hairy root transformation system in citron (*C. medica* L.), which produces prolific and rapid root growth in as little as 30–60 days (Figure 1a). Compared to other citrus species, hairy root induction in citron is much faster, with roots typically emerging around 2 weeks post‐transformation (Figure S1). GFP‐expressing roots could be readily visualized by fluorescent microscopy (Figure 1b,c). The transformation efficiency, as defined by the percentage of GFP‐positive roots among the total number of emerged roots, ranged from 25% to 73%. Further genotyping and expression analysis by PCR and RT‐PCR of *GFP*, *rolB*, and *rolC* marker genes confirmed the authenticity of the transgenic hairy roots (Figure 1d,e). As shown in Figure 1e, we detected robust expression of *GFP*, *rolB*, and *rolC* genes (all within the T‐DNA region) in the hairy root samples. No *virD* expression (a bacterial marker outside the T‐DNA region) was detectable in any of the samples or in the “No RT” controls, thus eliminating the interference of bacterial DNA contamination in the assays. When using *C*Las‐infected explants as a source for transformation, we detected systemic movement and accumulation of *C*Las in the hairy roots emerging from infected explants, but not in healthy explants, as confirmed by PCR using *C*Las‐specific primers (Figure 1d). Together, these results demonstrate that citron is highly amenable to hairy root transformation with high explant survival and transformation efficiency. Importantly, citron hairy roots also support *C*Las accumulation, which is important for downstream antimicrobial efficacy studies.

### Citron hairy roots can be utilized for rapid evaluation of antimicrobial genes

**Figure 2:**
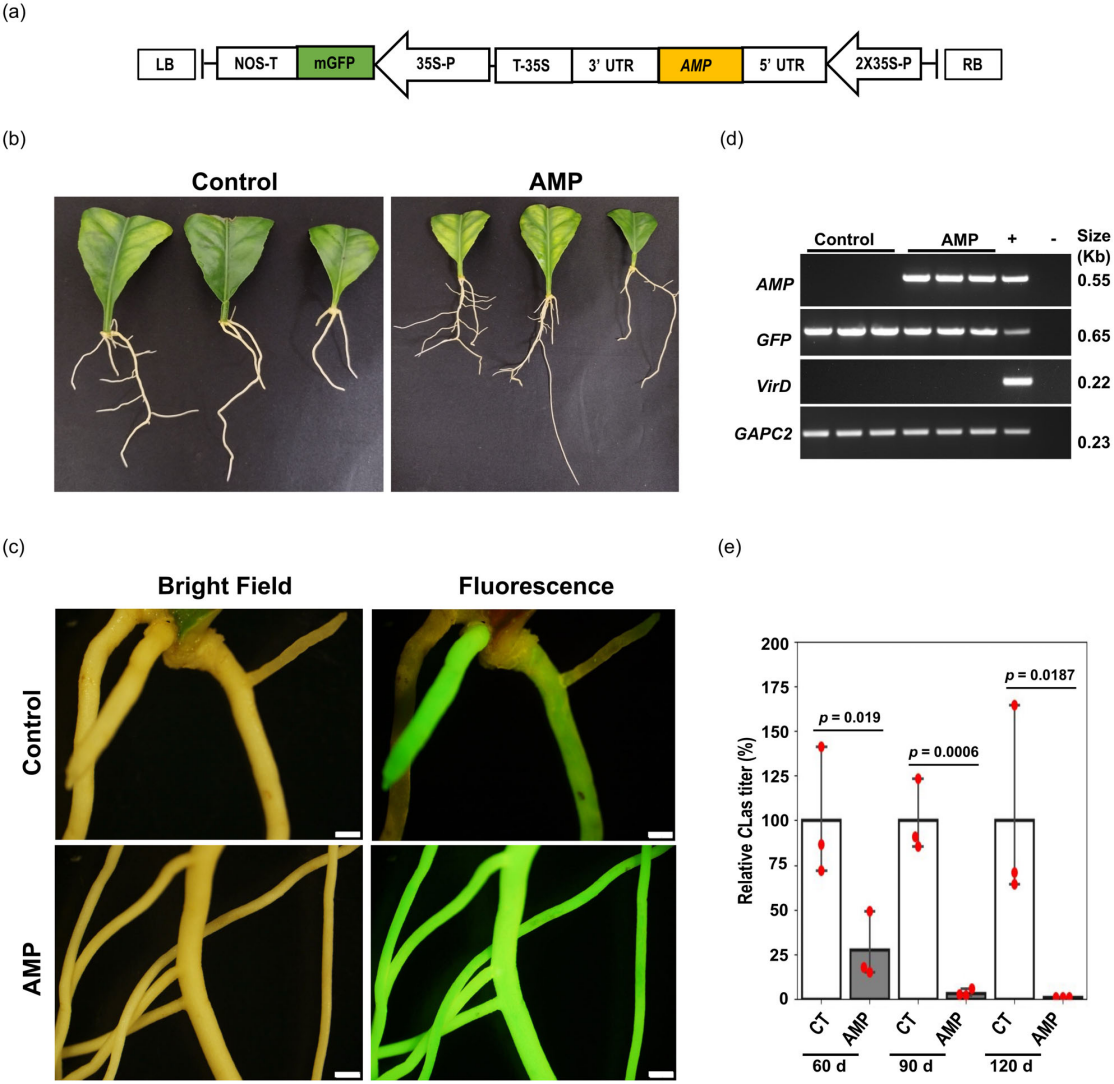
Effect of heterologous expression of spinach AMP on *Candidatus* Liberibacter asiaticus (*C*Las) titer in citron hairy roots. (a) A schematic diagram showing the *AMP* gene construct in a binary vector with GFP as a reporter. (b) Hairy roots induced by constructs with control (encoding GFP alone) and *AMP* (encoding both GFP and AMP) in citron leaves are shown. (c) Visualization of GFP expression in control and *AMP* transgenic hairy roots via fluorescence microscope. The images were taken at 60 days post‐transformation. Scale bars 1 mm. (d) Reverse transcription‐polymerase chain reaction (RT‐PCR) analysis to confirm the expression of *AMP* and *GFP* in transgenic hairy roots. *VirD*, a *Rhizobium rhizogenes* virulence gene, was used to assess bacterial contamination. “+” indicates a PCR positive control, and “−” is a no template control. *GAPC2*, an endogenous citrus gene, is used as a PCR control. Raw agarose gel images used to prepare panel D are shown in Figure S3. (e) Quantitative polymerase chain reaction (qPCR)‐based estimation of *C*Las titer in control and *AMP* overexpressing hairy roots at 60, 90, and 120 days post‐transformation. Error bars indicate the standard error of three independent biological replicates, and *P*‐values showing the significance of the difference are mentioned in the figure.

We previously demonstrated that specific antimicrobial peptides (AMPs) from spinach inhibit the accumulation of *C*Las and *C*Lso in citrus and potatoes, respectively (Irigoyen et al., 2020; Padilla et al., 2025). These AMPs disrupt bacterial cell membrane permeability, leading to cell mortality (Li et al., 2021; Padilla et al., 2025). Here, we evaluated the feasibility of the citron hairy root system for rapid efficacy testing of antimicrobial peptides. As proof‐of‐concept, we induced transgenic citron hairy roots using a vector control (encoding GFP alone) and an AMP construct (encoding both GFP and a spinach AMP) (Figure 2a,b). The transgenic roots were identified by GFP fluorescence under a fluorescence microscope (Figure 2c). Reverse transcription PCR (RT‐PCR) confirmed *AMP* and *GFP* expression in the hairy roots (Figure 2d). To assess *C*Las accumulation in transgenic roots, DNA was extracted from three independent biological replicates at 60, 90, and 120 d post‐transformation. Similar to our earlier research (Irigoyen et al., 2020), qPCR‐based quantification demonstrated that heterologous expression of spinach AMP significantly (*P* < 0.05 or 0.01) reduced *C*Las accumulation by ~72–99% in transgenic hairy roots (Figure 2e) compared to only vector‐transformed roots at all tested time points. These findings confirm that hairy root transformation in citron can be used to screen antimicrobial genes against HLB disease.

### Citron hairy roots can be utilized for rapid evaluation of CRISPR‐Cas9 gene targets

**Figure 3:**
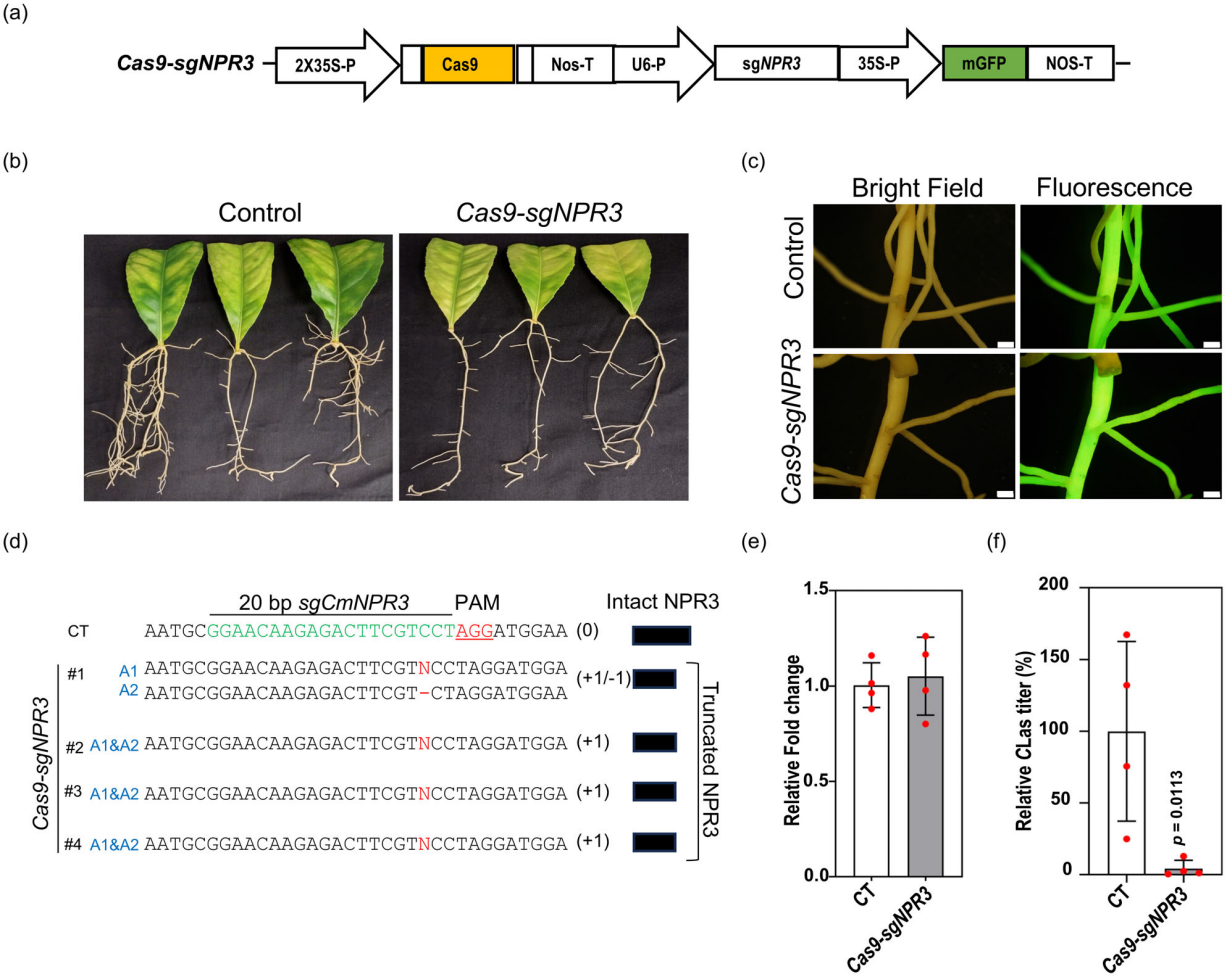
Effect of CRISPR‐Cas9‐mediated *NPR3* editing on *Candidatus* Liberibacter asiaticus (*C*Las) titer in citron hairy roots. (a) A schematic diagram showing the *Cas9‐sgNPR3* construct in a binary vector with GFP as a reporter. (b) Hairy roots were induced using the empty vector (control) carrying *GFP* and *Cas9* alone, and the *Cas9‐sgNPR3* vector carrying *GFP*, *Cas9*, and *sgNPR3*. (c) Confirmation of GFP expression in control and Cas9‐sgCmNPR3 transformed hairy roots via fluorescence microscope. The images were taken 60 days after transformation. Scale bars 1 mm. (d) Confirmation of *CmNPR3* editing in multiple independent hairy roots via amplicon sequence analysis. The 20 bp sgRNA, the PAM site, and the frameshift edits in the *CmNPR3* coding sequence in CT and *CmNPR3*‐edited hairy roots are indicated. (e) Reverse transcription‐quantitative polymerase chain reaction (RT‐qPCR)‐based expression analysis of the *NPR3* transcript in the control and *CmNPR3* edited hairy roots. (f) Estimation of *C*Las titer in control and *CmNPR3* edited hairy roots through qPCR. Error bars represent the standard error of four independent biological replicates, with the corresponding *P*‐value indicated in the figure.

We edited the citron *NPR3* (*CmNPR3*) gene using the CRISPR‐Cas9 system. NPR3 is reported as a negative regulator of plant defense response in potatoes (Ramasamy et al., 2024). However, its effect in supporting *C*Las proliferation in citrus is unknown. To assess this, we induced transgenic hairy roots from *C*Las‐infected leaves using either a vector control (encoding GFP and Cas9) or the sg*CmNPR3* CRISPR vector (encoding GFP, Cas9, and the sgRNA) (Figure 3a,b). At 60 days post‐transformation, multiple independent GFP‐positive hairy roots were collected for DNA extraction to confirm gene editing by Sanger DNA sequencing (Figure 3c,d). Multiple frameshift mutations (biallelic heterozygous and monoallelic homozygous) were identified among the edited roots (Figure 3d). Four biological replicate samples were subsequently used for estimating the expression of *NPR3* and levels of *C*Las. The genomic‐level edits had no impact on the *NPR3* transcript levels (*P* = 0.704) compared to the control roots (Figure 3e). This was not unexpected, given that frameshift mutations were resulting from single‐nucleotide insertions or deletions (InDels) (Figure 3d). The *C*Las titer estimation revealed a significant (>95%, *P* = 0.01) reduction in *C*Las accumulation in the *NPR3* edited roots compared to the control (Figure 3f). Our results indicate that the *NPR3* gene in citrus can be modulated to develop tolerance against HLB disease.

### Citron hairy roots can be utilized for rapid efficacy testing of antimicrobial chemicals

**Figure 4:**
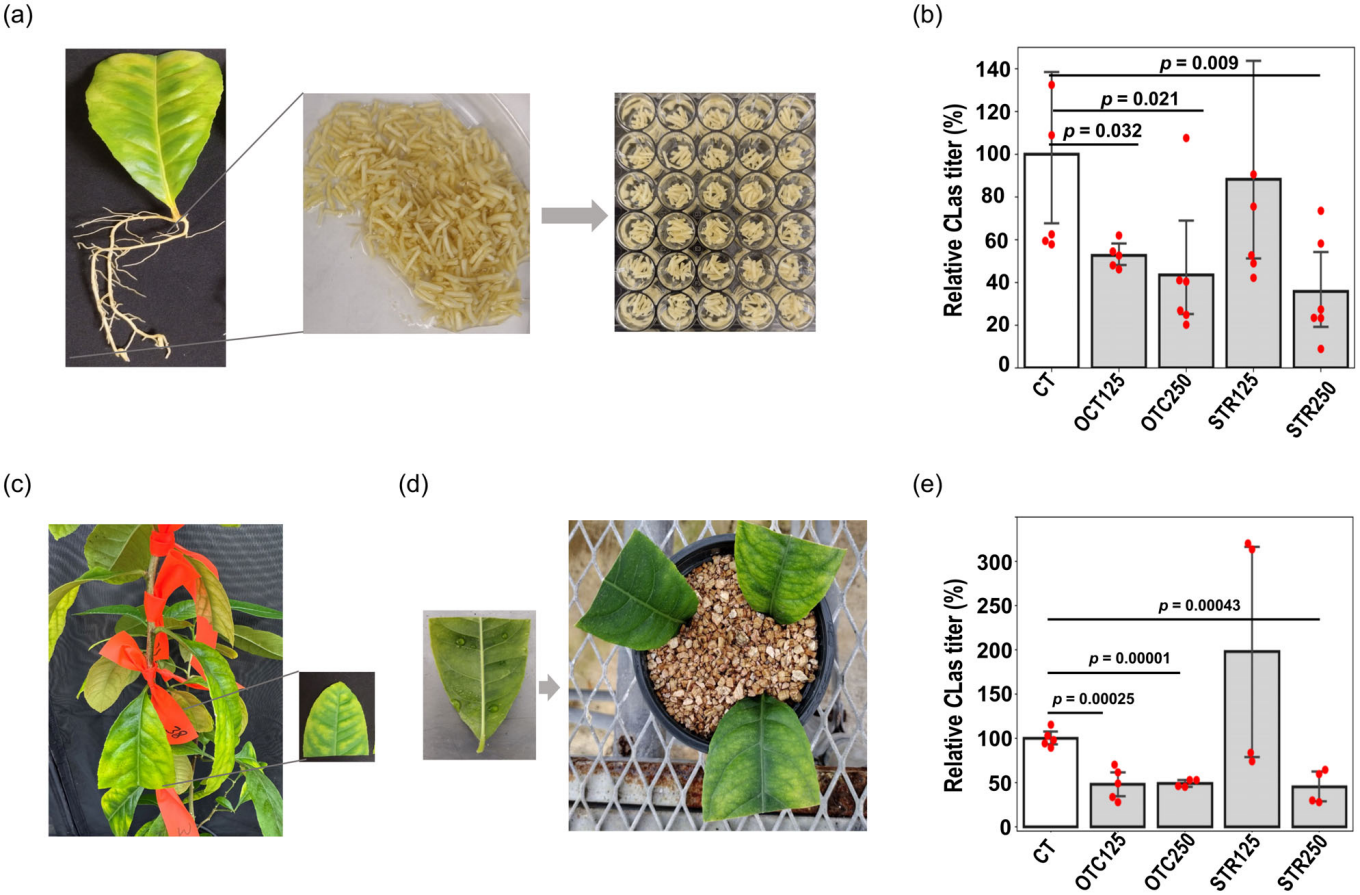
Citron hairy roots and leaf infiltration assays for antimicrobial screening. (a) A representative photograph shows the pooled root tissues from multiple explants used for the hairy root assay. (b) The relative percentage of *Candidatus* Liberibacter asiaticus (*C*Las) titer in the hairy roots of the untreated control (CT) and those treated with different antibiotics was estimated via quantitative polymerase chain reaction (qPCR). Error bars represent the standard error of five to six biological replicates, with their respective *P*‐values shown above. (c) A photograph showing leaf tagging on the citron tree. The lower half‐leaf portions were used to estimate *C*Las titer via qPCR. (d) Infiltration of water or different antibiotics in detached half leaves (lower half) and further incubation in vermiculite. (e) The relative percentage of *C*Las titer in CT and antibiotics‐treated leaves was estimated via qPCR. Error bars represent the standard error of four to five biological replicates, with their respective *P*‐values shown above.

Different genetic engineering approaches may be used to develop HLB resistance/tolerance in citrus. However, it is a lengthy process that requires numerous federal regulatory approvals. Alternatively, as a short‐term solution, *C*Las‐citrus hairy roots can be used to rapidly screen various antimicrobials against HLB disease (Irigoyen et al., 2020). Here, as proof‐of‐concept, we showed the utility of citron hairy roots for efficacy testing and screening of antimicrobial compounds using reference *C*Las inhibitors, oxytetracycline (OTC) and streptomycin (STR). The hairy roots were induced from *C*Las‐infected leaves (Figure 4a), validated for *C*Las presence by quantitative PCR (qPCR), and subsequently used to conduct efficacy assays *in vitro* in a 48‐well plate. Five to six biological replicate hairy root samples were treated with OTC and STR, along with untreated control samples (CT). After 72 h of incubation, the roots were treated with propidium monazide‐based reagent (PMAxx) to measure viable *C*Las titers (Irigoyen et al., 2020). As expected, the qPCR‐based molecular diagnostics revealed that OTC at 125 (52.65%, *P* < 0.05) and 250 PPM (43.5%, *P* < 0.05), and STR at 250 PPM (35.79%, *P* < 0.01) significantly inhibited *C*Las titer compared to untreated control (CT) (Aksenov et al., 2024; Vieira et al., 2025) (Figure 4b). The results confirm that the citron hairy roots assay can be used for faster and scalable screening of many antimicrobials against *C*Las bacterium.

### Citron leaves can be used for transient leaf infiltration assays and efficacy studies

We also examined the effect of OTC and STR using an excised leaf infiltration assay. The antibiotics were infiltrated directly into *C*Las‐infected citron leaves (Figure 4c,d), and DNA was extracted after 10 days post‐infiltration. Similar to the hairy root assay, qPCR‐based quantification revealed that compared to control (water infiltration), OTC infiltration at both 125 (48.52%, *P* < 0.01) and 250 PPM (49.18%, *P* < 0.01), and STR infiltration at 250 PPM (45.29%, *P* < 0.01) significantly reduced *C*Las accumulation (Figure 4e).

## DISCUSSION

HLB, a devastating bacterial disease, is a major threat to citrus productivity worldwide. Almost all citrus species are susceptible to *C*Las with varying symptoms, suggesting a complex nature of the disease's etiology. The currently available treatments include planting disease‐free citrus germplasms, selectively removing HLB‐infected trees, breaking HLB transmission by the psyllid vector, and enhancing the plant's immunity (Graham et al., 2024). There is an urgent need to identify both long‐term and immediate solutions to control the adverse effects of HLB disease. However, due to the fastidious nature of the *C*Las, recalcitrance, and the hardy nature of citrus trees, strategies for screening new antimicrobials using conventional whole‐plant applications in a greenhouse or field are not high‐throughput. Additionally, genetic screening for gain‐ or loss‐of‐function targets using *Agrobacterium*‐based stable transformation or viral vector‐based transient expression is laborious and time‐consuming. Furthermore, the local application of antimicrobial drugs/compounds to infected citrus plants, such as trunk injections and foliar applications, poses challenges due to a lack of precise delivery into the phloem tissues, resulting in local treatment of the infected plants (Kennedy et al., 2023). We previously demonstrated that hairy roots induced using grapefruits and oranges can be useful for efficacy testing new antimicrobials against HLB. However, these cultivars still required a substantial amount of time (~90–120 days) to produce hairy roots (Irigoyen et al., 2020). In this study, we optimized a significantly improved version of the hairy root transformation system based on the citron cultivar. The improved protocol enables the rapid induction and robust growth of hairy roots in citron (~30–60 days), compared to grapefruits and oranges (90–120 days) (Figure S1), supporting multiple downstream genetic and chemical evaluations.

The utility of the citron‐based hairy root system for genetic screening was further demonstrated by evaluating the efficacy of an antimicrobial peptide (AMP). The AMP expression in citron hairy roots progressively decreased *C*Las accumulation in the hairy roots (Figure 2). Furthermore, we evaluated the function of a known gene involved in plant defense signaling and its effects on the survival of the *C*Las bacterium. The *NPR* gene family comprises multiple members and regulates salicylic acid‐mediated plant defenses. The antagonistic activity of *NPR1* as a positive defense regulator and *NPR3* as a negative defense regulator is well characterized (Ali et al., 2017; Backer et al., 2019). In this study, we examined the loss‐of‐function effects of *CmNPR3* on *C*Las using the CRISPR‐Cas9 approach in the optimized hairy root system. We confirmed the editing of *CmNPR3* in the hairy roots, resulting in frameshift mutations in the *NPR3* coding sequence (Figure 3d). The *NPR3* transcript level remained unchanged in the *NPR3*‐edited roots when compared to controls. This is not uncommon for single‐ to a few‐nucleotide frameshift genetic mutations, but it could result in truncated proteins and potentially a dysfunctional NPR3 protein (Irigoyen et al., 2020; Ramasamy et al., 2024). Notably, qPCR‐based quantification revealed a significant reduction in *C*Las accumulation in the *CmNPR3*‐edited citron hairy roots. A recent study analyzed the function of *NPR3* in *C. sinensis* and associated it with callus deposition and accumulation of ROS; however, the effect on *C*Las titer was not assessed (Sarkar et al., 2024). Our hairy root bioassay results suggest that gene editing of *CmNPR3* can lead to a decrease in the accumulation of *C*Las in citrus. Lastly, the citron hairy roots can be used to rapidly screen antimicrobial compounds (Figure 4). In this approach, antimicrobial compounds are directly added to the culture media containing citron hairy roots. After 72 h, the titer of the *C*Las can be quantified using quantitative PCR (qPCR). We evaluated the efficacy of reference antibiotics (OTC and STR) using the citron hairy root assay, and the results show a dose‐dependent effect of the antibiotics on *C*Las accumulation.

In conclusion, our optimized citron‐based hairy root system enables the rapid efficacy screening of diverse antimicrobial compounds and biotechnological targets, thereby accelerating the discovery of potential HLB therapies. It will serve as a valuable resource for the HLB research community.

## MATERIALS AND METHODS

### Citron hairy root transformation

An *ex vivo* approach was used to induce hairy roots from healthy and *C*Las‐infected citron explants. A recombinant *R. rhizogenes* (ATCC^®^ 43056™) harboring a GFP reporter plasmid (pBin‐mGFP) was grown in a virulence induction medium (VIM) containing glucose (1%), 2‐morpholinoethanesulfonic acid (MES, 74.8 mM), sodium phosphate buffer (1.2 mm, pH 5.6), acetosyringone (100 μm), and 1× concentration of 20× AB salts consisting of NH_4_Cl (20 g/L), MgSO_4_.7H_2_O (6 g/L), KCl (3 g/L), CaCl_2_.2H_2_O (0.264 g/L) and FeSO_4_.7H_2_O (0.05 g/L). The bacterial culture (OD_600_ = 0.8) was pelleted and resuspended in 50 ml of freshly prepared Murashige and Skoog (MS) medium containing acetosyringone (100 μm), spermidine (1 mm), and lipoic acid (5 μm). The culture was then incubated at room temperature (25°C) on a rotatory shaker (Thermo Fisher Scientific, Waltham, MA, USA) at 50 rpm for 2 h. To prepare explants, sections of ~5 cm from the citron branch, with a single node and leaves, were excised from the mother plant. The bacterial suspension was pelleted down for infiltration, and the cut side of the explants was gently touched with the bacterial pellet. The explants were then carefully transferred to a wet vermiculite matrix and placed in a greenhouse with frequent misting irrigation (Figure 1a). The transgenic roots were identified by GFP fluorescence using a stereo microscope (Olympus Corporation, Hachioji, Tokyo, Japan) (Figure 1b,c). The transformation efficiency was determined using the formula [number of GFP‐positive roots/total number of roots] × 100. To confirm the authenticity of the transgenic hairy roots, RNA was extracted using the Direct‐zol RNA Miniprep Kit (Zymo Research, Irvine, CA, USA), which included a DNase treatment step to eliminate DNA contamination. Plant‐specific cDNAs were synthesized using an oligo(dT) primer. Expression of *GFP* present on the binary T‐DNA and *rolB, rolC* present on the Ri T‐DNA (Triplett et al., 2008), was determined by RT‐PCR. To examine possible bacterial DNA contamination in RT‐PCR, a *R. rhizogenes* virulence gene, *VirD*, which is present on the Ri plasmid but outside of the Ri T‐DNA region, was used as a diagnostic marker (Haas et al., 1995). The presence of *C*Las in hairy roots was confirmed by PCR amplification of the ribosomal protein gene (*rplA*, A2‐F/J5‐R) (Hocquellet et al., 1999). A citrus gene encoding glyceraldehyde‐3‐phosphate dehydrogenase C2 (*GAPC2*) was used as a PCR control (Irigoyen et al., 2020) (Figure 1d,e; Table S1).

### Efficacy testing of antimicrobial peptides (AMP) using citron hairy roots

For AMP transformation, the *R. rhizogenes* harboring an antimicrobial peptide (*AMP*) construct or empty vector (pBin‐mGFP) was used to induce hairy roots from leaf explants (Figure 2a). Prior to transformation, the *C*Las presence in leaf explants was confirmed by qPCR (Table S2). After 60, 90, and 120 days post‐transformation, three independent biological replicate samples of GFP‐positive hairy roots were collected. Note that users can choose to sample hairy roots earlier than 60 days, depending on the amount of root needed for the downstream assay. Because *C*Las titer and natural distribution are highly uneven in citrus roots (Louzada et al., 2016), independent GFP‐positive hairy roots from multiple explants were randomly sampled for the three biological replicates. The AMP expression in hairy roots was examined by RT‐PCR analysis using 5′ and 3′ untranslated regions (UTRs) primers (TEV‐F and TEV‐R) of *Tobacco etch virus* (*Potyvirus nicotianainsculpentis*) flanking the AMP gene (Figure 2d, Table S1). *C*Las accumulation in hairy roots was estimated by qPCR using 25 ng of extracted DNA and iTaq™ Universal SYBR® Green Supermix in a CFX384 Real‐Time PCR (Bio‐Rad Laboratories, Hercules, CA, USA). The ribonucleotide reductase β‐subunit primers (RNRf/RNRr) were used to detect *C*Las, while citrus *GAPC2* was used for normalization (Figure 2e) (Mafra et al., 2012; Zheng et al., 2016).

### CRISPR‐Cas9‐mediated *CmNPR3* editing in citron hairy roots

An endogenous *NPR3* gene in citron (*CmNPR3*; Cm196830) was identified by blasting Arabidopsis NPR3 (*AtNPR3*; AT5G45110) against the citrus genome database (https://www.citrusgenomedb.org/). A *CmNPR3*‐specific single guide RNA (sg*CmNPR3*; GGAACAAGAGACTTCGTCCT) was predicted using the CRISPR‐P 2.0 tool (Liu et al., 2017). The CRISPR construct (Cas9:sg*CmNPR3*:mGFP) and empty vector were transformed into *R. rhizogenes* to induce hairy roots from *C*Las‐infected leaves (Table S3). 60 days after transformation, GFP‐positive hairy roots were collected. Multiple independent hairy roots were sampled and subjected to amplicon sequencing. The genomic DNA was extracted from independent hairy roots, and the 550 bp of *CmNPR3* was PCR amplified with gene‐specific primers flanking the target region (Table S1) using Q5® High‐Fidelity DNA Polymerase (New England Biolabs, Ipswich, MA, USA). The independent PCR amplicons were sequenced (EtonBio Science, San Diego, CA, USA), and DNA mutations in *CmNPR3* were identified using the Synthgo ICE tool (Conant et al., 2022). Based on the amplicon sequencing data, we observed an editing efficiency of greater than 90%, resulting in multiple frameshift mutations in the *NPR3* coding sequence, which could lead to truncated NPR3 (Figure 3d). We then proceeded to quantification of *NPR3* expression and *C*Las using RT‐qPCR and qPCR analysis, respectively, using four biological replicate samples (Figure 3f). To quantify *NPR3* transcript levels, total RNA was extracted from control and edited roots, and cDNA was synthesized from 1 μg of RNA. NPR3‐specific primers were designed (Table S1), and *NPR3* expression in control and edited hairy roots was quantified by RT‐qPCR using iTaq™ Universal SYBR® Green Supermix in a CFX384 Real‐Time PCR system (Bio‐Rad Laboratories). Citrus *GAPC2* was used as the reference gene for normalization.

### Efficacy testing of antimicrobial chemicals using citron hairy roots

The *C*Las‐infected hairy roots were surface sterilized using 70% ethanol for 30 seconds, followed by 2.5% bleach for 10 min, and then rinsed thoroughly with autoclaved distilled water. The roots were cut into small pieces, and 100 mg of pooled roots were transferred individually into a 48‐well plate (Figure 4a). The wells were then filled with either Gamborg's B‐5 medium or B5 supplemented with antibiotics [oxytetracycline (OTC) or streptomycin (STR) at concentrations of 125 PPM and 250 PPM]. Five to six biological replicates were used for treatments and untreated controls. The plates were then vacuum‐infiltrated at ~20 psi for 30 min and incubated on a 3D platform rotator (Thermo Fisher Scientific) at 50 rpm for 72 h. After antibiotic treatments, the roots were treated with 50 μm PMAxx™ dye (Biotium, Fremont, CA, USA) (a DNA‐binding dye used to distinguish live from dead bacterial cells in qPCR assays) in the dark while rotating at 50 rpm for 15 min. The samples were then crosslinked using a Glo‐Plate Blue LED Illuminator for 20 min. Total DNA was then extracted from the control, OTC‐ and STR‐treated root samples, and *C*Las titer was quantified by qPCR using 25 ng of template DNA, as described above. The relative bacterial accumulation was plotted in different treatments (Figure 4b).

### Citron leaf infiltration bioassay

Since *C*Las distribution in citrus plants is uneven, citron leaves were first tagged to identify *C*Las‐infected tissues (Figure 4c). The upper half of each tagged leaf was used for DNA extraction and quantification of *C*Las titer by qPCR. Four to five biological replicates were used for treatments and untreated controls. The lower halves of *C*Las‐positive leaves (Table S4) were detached and pierced at two locations. A 1 ml needleless syringe, filled with either 500 μl of water or an antibiotic solution (OTC or STR) at concentrations of 125 and 250 ppm, was positioned over the pierced site. The leaves were gently and completely infiltrated (Figure 4d). The infiltrated leaves were then transferred to a vermiculite matrix and placed in a greenhouse with frequent water misting (Figure 4d). After 10 days post‐infiltration, DNA was extracted from the leaves. The relative *C*Las titer was quantified by qPCR in different treatments (Figure 4e).

## AUTHOR CONTRIBUTIONS

AK, MR, CKT, JB, and KM contributed to the experimental design. AK, MR, SI, CKT, and CP conducted the experiments and analyzed the data. JB and KM supervised the experiments. All authors contributed to the preparation and review of the manuscript.

## CONFLICT OF INTEREST

The authors declare that the research was conducted in the absence of any commercial or financial relationships that could be construed as a potential conflict of interest.

## Supporting information


**Figure S1.** *Rhizobium rhizogenes*‐mediated hairy root transformation in different citrus cultivars. The approximate timeline for hairy root induction in citron begins at 14 days, and sufficient root growth can be achieved by 60 days, allowing for the collection of multiple biological replicates for various downstream assays. In contrast, it takes approximately 90–120 days for the induction and growth of hairy roots in grapefruit and sour oranges.
**Figure S2.** Raw agarose gel images used to prepare Figure 1d (a) and Figure 1e (b).
**Figure S3.** Raw agarose gel images used to prepare Figure 2d.

**Table S1.** List of primers used in this study.
**Table S2.** Table showing *Candidatus Liberibacter asiaticus* (*C*Las) titer (normalized Ct) in leaf explants used to induce hairy roots by vector control and an AMP overexpressing construct.
**Table S3.** Table showing *Candidatus Liberibacter asiaticus* (*C*Las) titer (normalized Ct) in leaf explants used to induce hairy roots by vector control and *CmNPR3* CRISPR construct.
**Table S4.** Table showing *Candidatus Liberibacter asiaticus* (*C*Las) titer (normalized Ct) in leaf explants used for antibiotics infiltration.

## Data Availability

All the data presented in this study are included in the main article and Supporting Information. Further inquiries can be directed to the corresponding author.
