# Supplementary material for: High‐efficiency genome‐editing, transgene evaluation, and antimicrobial efficacy testing using Citrus medica L. hairy roots
Source: Plant J. 2026 Feb 18;125(4):e70745. doi: 10.1111/tpj.70745 (PMC12917295; doi:10.1111/tpj.70745)
Supplement: Supplementary file 1 — Figure S1. Rhizobium rhizogenes‐mediated hairy root transformation in different citrus cultivars. The approximate timeline for hairy root induction in citron begins at 14 days, and sufficient root growth can be achieved by 60 days, allowing for the collection of multiple biological replicates for various downstream assays. In contrast, it takes approximately 90–120 days for the induction and growth of hairy roots in grapefruit and sour oranges. Figure S2. Raw agarose gel images used to prepare Figure 1d (a) and Figure 1e (b). Figure S3. Raw agarose gel images used to prepare Figure 2d. [file TPJ-125-0-s002.pdf]

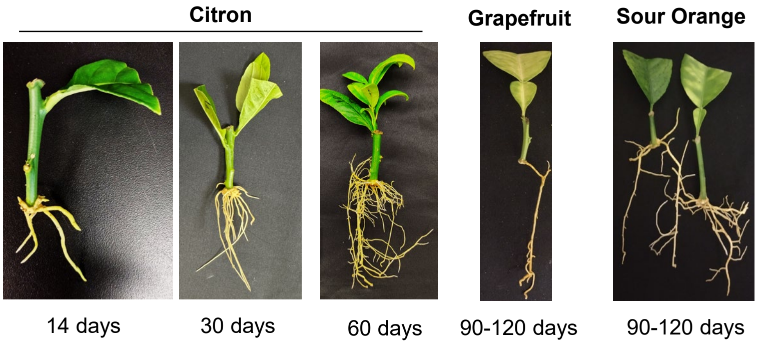

Supplementary Figure 1. *Rhizobium rhizogenes*-mediated hairy root transformation in different citrus cultivars. The approximate timeline for hairy root induction in citron begins at 14 days, and sufficient root growth can be achieved by 60 days, allowing for the collection of multiple biological replicates for various downstream assays. In contrast, it takes approximately 90-120 days for the induction and growth of hairy roots in grapefruit and sour oranges.

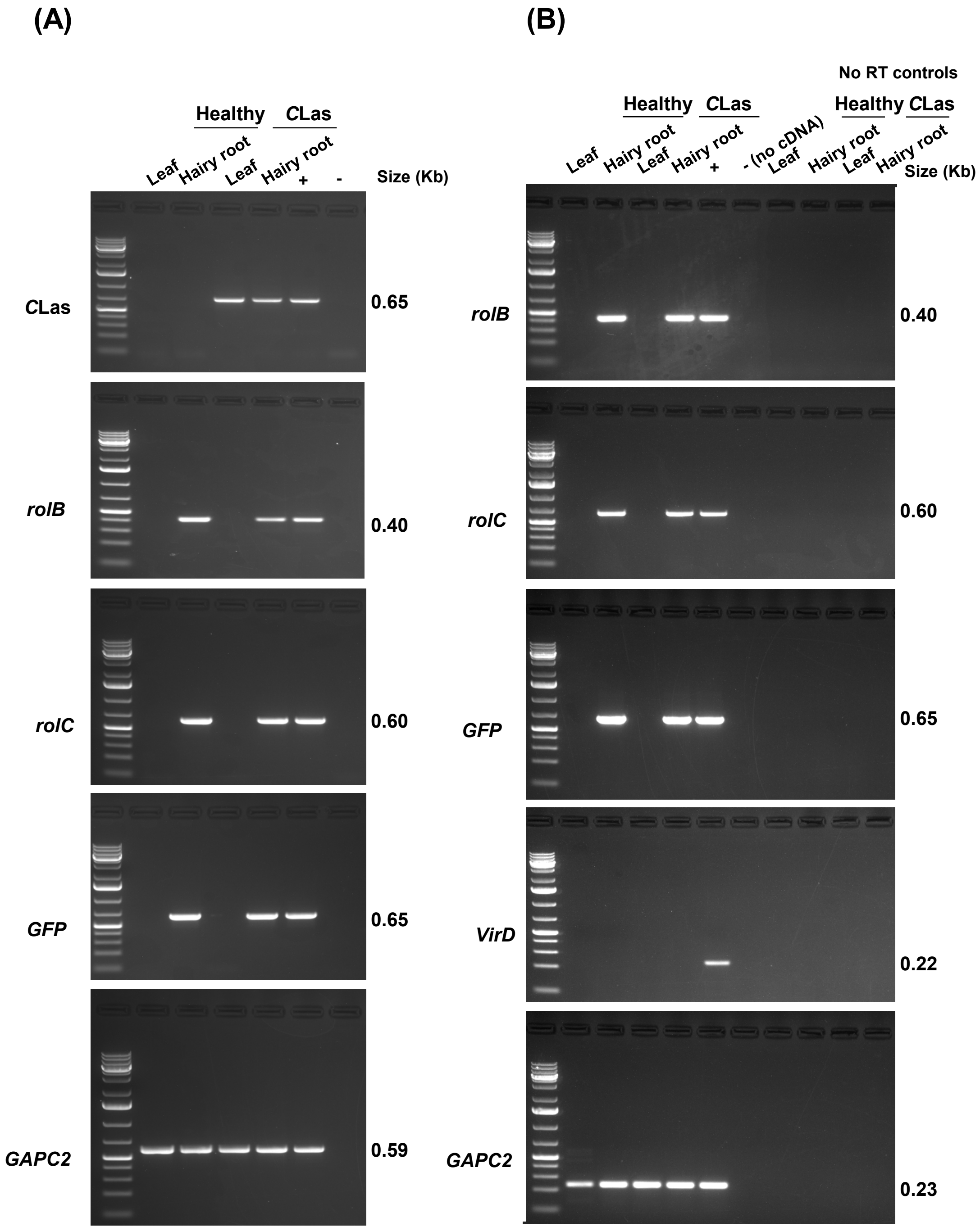

Supplementary Figure 2. Raw agarose gel images used to prepare Fig. 1D (A) and Fig. 1E (B)

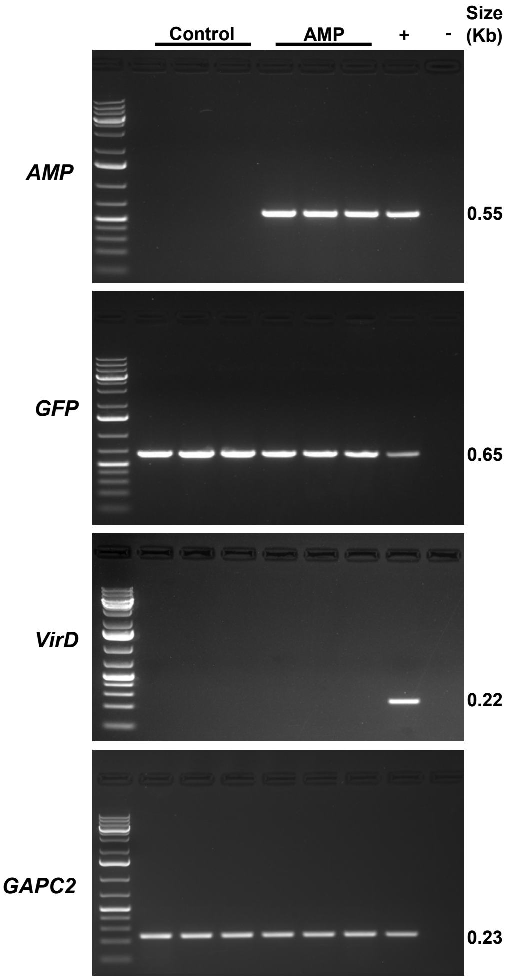

Supplementary Figure 3. Raw agarose gel images used to prepare Fig. 2D
